# A Live *Salmonella* Vaccine Delivering PcrV through the Type III Secretion System Protects against Pseudomonas aeruginosa

**DOI:** 10.1128/mSphere.00116-19

**Published:** 2019-04-17

**Authors:** Julia Aguilera-Herce, Meritxell García-Quintanilla, Rocío Romero-Flores, Michael J. McConnell, Francisco Ramos-Morales

**Affiliations:** a Departamento de Genética, Facultad de Biología, Universidad de Sevilla, Seville, Spain; b Institute of Biomedicine of Sevilla (IBiS), University Hospital Virgen del Rocío/CSIC/Universidad de Sevilla, Seville, Spain; c Antimicrobial Resistance and Hospital Acquired Infections Reference Laboratory, National Centre for Microbiology, Instituto de Salud Carlos III, Majadahonda, Spain; University of Maryland School of Medicine

**Keywords:** PcrV, Pseudomonas aeruginosa, *Salmonella*-delivered vaccines, SseJ, type III secretion

## Abstract

Pseudomonas aeruginosa is a common Gram-negative opportunistic pathogen that is intrinsically resistant to a wide range of antibiotics. The development of a broadly protective vaccine against P. aeruginosa remains a major challenge. Here, we used an attenuated strain of Salmonella enterica serovar Typhimurium as a vehicle to express P. aeruginosa antigens. A fusion between the S. enterica type III secretion effector protein SseJ and the P. aeruginosa antigen PcrV expressed under the control of the *sseA* promoter was translocated by *Salmonella* into host cells *in vitro* and elicited the generation of specific antibodies in mice. Mice immunized with attenuated *Salmonella* expressing this fusion had reduced bacterial loads in the spleens and lungs and lower serum levels of proinflammatory cytokines than control mice after P. aeruginosa infection. Importantly, immunized mice also showed significantly enhanced survival in this model. These results suggest that type III secretion effectors of S. enterica are appropriate carriers in the design of a live vaccine to prevent infections caused by P. aeruginosa.

**IMPORTANCE** The Gram-negative bacterium Pseudomonas aeruginosa is an important opportunistic pathogen that causes infections in cystic fibrosis and hospitalized patients. Therapeutic treatments are limited due to the emergence and spread of new antibiotic-resistant strains. In this context, the development of a vaccine is a priority. Here, we used an attenuated strain of Salmonella enterica serovar Typhimurium as a vehicle to express and deliver the *Pseudomonas* antigen PcrV. This vaccine induced the generation of specific antibodies in mice and protected them from lethal infections with P. aeruginosa. This is an important step toward the development of an effective vaccine for the prevention of infections caused by P. aeruginosa in humans.

## Abstract (precis)

The Gram-negative bacterium Pseudomonas aeruginosa is an important opportunistic pathogen that causes infections in cystic fibrosis and hospitalized patients. Therapeutic treatments are limited due to the emergence and spread of new antibiotic-resistant strains. In this context, the development of a vaccine is a priority. Here, we used an attenuated strain of Salmonella enterica serovar Typhimurium as a vehicle to express and deliver the *Pseudomonas* antigen PcrV. This vaccine induced the generation of specific antibodies in mice and protected them from lethal infections with P. aeruginosa. This is an important step toward the development of an effective vaccine for the prevention of infections caused by P. aeruginosa in humans.

## INTRODUCTION

Pseudomonas aeruginosa is an environmentally ubiquitous, Gram-negative, opportunistic bacterial pathogen. It is one of the more commonly reported nosocomial pathogens (1). P. aeruginosa forms biofilms in the upper airways of cystic fibrosis patients and repeatedly colonizes the lower airways, leading to chronic lung infection (2). It is also a common pathogen linked to burn wound infections (3), ventilator-associated pneumonia in intensive care unit patients (4), and urinary infections in patients with catheters in the upper urinary tract (5). In addition, P. aeruginosa is a leading cause of life-threatening infections in immunocompromised hosts with underlying diseases such as cancer or AIDS (6).

P. aeruginosa is intrinsically resistant to a wide range of antibiotics (7) and possesses adaptation strategies that facilitate its persistence in the environment, such as biofilm formation (8). In addition, the increasing selection of additional acquired resistance mechanisms, via mutations or horizontal gene transfer, has led to the emergence of multidrug-resistant strains (9).

In this context, the development of vaccines that limit the spread of P. aeruginosa infections is a major challenge. This has been the focus of research efforts for almost half a century, and over the last 25 years, multiple P. aeruginosa vaccines have been assessed in clinical trials (10). However, with the recent failure of the IC43 vaccine in a phase II clinical trial (11), there are currently no approved vaccines against P. aeruginosa or vaccines in advanced stages of clinical development (12). Renewal of the P. aeruginosa vaccine pipeline is thus a high priority.

Many antigens and delivery protocols have been tested as vaccine candidates, but to increase the efficacy of vaccination, novel approaches are clearly needed. Such approaches may combine previously tested P. aeruginosa antigens with delivery methods that were successful for other antigens.

The protective efficacy of outer membrane proteins OprF and OprI have been shown in animal models and clinical trials (13, 14). Another promising candidate is the P. aeruginosa V antigen (PcrV), the tip protein of the type III secretion system (T3SS), which is critical for its assembly and regulation. These secretion systems are present in many Gram-negative pathogens and symbionts and inject effector proteins into host cells to interfere with host cellular processes (15). Blockade of PcrV by specific antibodies inhibits the translocation of type III effector proteins, and immunization with recombinant PcrV or administration of anti-PcrV antibodies can protect animals from lethal P. aeruginosa infections (16, 17). Killed whole-cell and live attenuated P. aeruginosa vaccines present multiple antigens to the immune system but may exhibit some toxicity or residual virulence, whereas the use of recombinant proteins is safer but may induce a weaker immune response (18).

The use of live attenuated bacterial or viral pathogens is an interesting alternative for delivering heterologous antigens (19). *Salmonella* is among the first bacteria used for this purpose and has well-established protocols for genetic manipulation. Additional advantages of *Salmonella*-based vaccines are the low cost of production, absence of animal products, safety, and elicitation of efficient humoral and cellular immune responses via stimulation of innate and adaptive immunity (20). Salmonella enterica is a facultative intracellular pathogen that, once inside the host cell, resides in a modified phagosome known as the *Salmonella*-containing vacuole (SCV) (21). S. enterica possesses two T3SSs, T3SS1 and T3SS2, encoded in *Salmonella* pathogenicity islands 1 (SPI1) and 2 (SPI2), respectively (22–24). T3SS1 translocates effector proteins through the host plasma membrane and is required for invasion of nonphagocytic cells. T3SS2 is necessary for intracellular survival and secretes effectors from inside the SCV. Previous studies have shown that T3SS-mediated translocation can be used for efficient delivery of heterologous antigens that are fused to effector proteins to the cytosol of antigen-presenting cells (25–27). In fact, live S. enterica vaccines based on these delivery systems have been shown to produce protective immunity against other bacterial and viral pathogens (28–31) and are also being used in the field of tumor immunotherapy (32–36).

The aim of this study was to develop a vaccine against P. aeruginosa using S. enterica T3SS effectors as carriers for relevant antigens. A strong protective immune response against the PcrV antigen was obtained using the SseJ effector as a carrier.

## RESULTS

### Construction and evaluation of *Salmonella* vectors for delivery of P. aeruginosa antigens.

**FIG 1:**
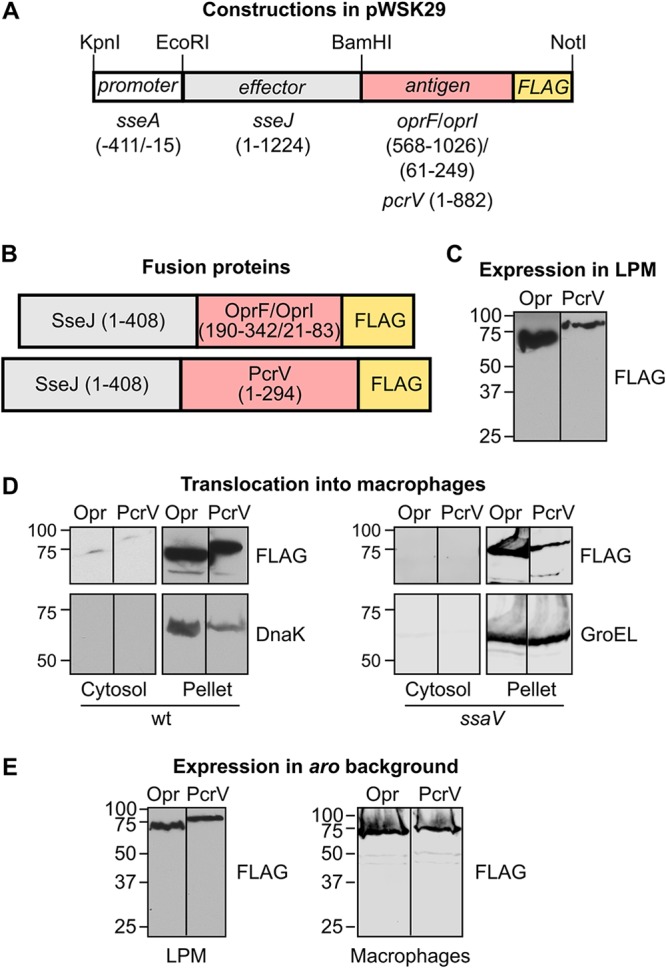
Generation of protein fusions for delivery of Pseudomonas aeruginosa antigens. (A) Vector pWSK29 was used for the generation of two plasmids in three steps. In the first step, part of the coding region of antigens OprF/OprI (see Materials and Methods for the generation of the hybrid gene *oprF-oprI*) or the complete coding region (without the stop codon) for the antigen PcrV from P. aeruginosa was amplified by PCR. DNA encoding the FLAG epitope and recognition sites for BamHI and NotI endonucleases was added with the primers used for amplification. These amplicons were cleaved with BamHI and NotI and ligated into pWSK29 previously cleaved with the same restriction enzymes. In the second step, the coding region of *sseJ* without the stop codon was amplified by PCR and ligated to the plasmids obtained in the first step previously cleaved with EcoRI and BamHI restriction enzymes. Finally, the promoter region of *sseA* (nucleotides −411 to −15 relative to the translation start site) was amplified by PCR and added to the plasmids obtained in the second step previously cleaved with endonucleases KpnI and EcoRI. Numbers in parentheses indicate base pairs relative to the start of the coding region. (B) Fusion proteins that are expected to be produced under the appropriate conditions after the introduction of the plasmids described in panel A in *S.* Typhimurium. Numbers in parentheses refer to amino acids included in the fusions. Both fusions include the complete amino acid sequence of SseJ and a C-terminal FLAG epitope to facilitate identification of the fusions by Western blotting. The expected molecular masses are 70.18 kDa for SseJ-OprF/I-FLAG and 79.23 kDa for SseJ-PcrV-FLAG. (C) Derivatives of *S.* Typhimurium strain 14028 carrying plasmids encoding OprF/I (Opr) or PcrV fusion proteins were grown in LPM. A FLAG tag was used for detection of the proteins by immunoblotting. Molecular weights in kDa are indicated on the left. (D) *Salmonella* strains that were derivatives of the wild-type strain (wt) or the *ssaV* mutant (carrying a nonfunctional T3SS2) expressing SseJ in fusion with *Pseudomonas* antigens OprF/I (Opr) or PcrV with a FLAG tag were grown under noninvasive conditions. These bacteria were used to infect RAW264.7 cells for 8 h. Cells were lysed with 1% Triton X-100 in PBS and centrifuged 15 min at 15,000 × *g*. The pellets and the filtered concentrated supernatants (cytosol) were analyzed by immunoblotting with anti-FLAG antibodies to detect translocation of fusion proteins into the host cytosols. Incubations with anti-DnaK or anti-GroEL antibodies were used as controls of contamination of the cytosol fraction with nonsecreted bacterial proteins. (E) Expression of the fusion proteins SseJ-OprF/I (Opr) and SseJ-PcrV (PcrV) was analyzed in an *aroA aroB* (*aro*) background. Bacteria were grown in LPM (left) or were grown for 24 h at 37°C with shaking in LB medium and used to infect RAW264.7 macrophages (right). Protein extracts were analyzed by immunoblotting with an anti-FLAG antibody.

A previous study showed that the effector SseJ was an appropriate carrier for inducing a robust immune response against the model antigens ovalbumin and listeriolysin *in vitro* and *in vivo* (25). We used the promoter of the *Salmonella* SPI2 gene *sseA* (P*sseA*) to direct the expression of fusion proteins containing the *Salmonella* effector SseJ as well as *Pseudomonas* antigens OprF/I and PcrV (Fig. 1A and B and Materials and Methods). SseJ is secreted through the T3SS2 from inside the SCV (37), and P*sseA* responds to intravacuolar signals and is induced several hours after invasion (38, 39). The constructs were prepared using the low-copy-number plasmid pWSK29 as the vector. S. enterica serovar Typhimurium strain 14028 containing the plasmids with the hybrid genes was grown in low-magnesium minimal medium (LPM), a medium that imitates intravacuolar conditions, and the expression of fusion proteins was monitored by Western blotting against the FLAG tag that was also added. The results shown in Fig. 1C indicate that both plasmids yielded significant levels of protein production. Strains containing these plasmids were tested for their capacity to translocate the fusion proteins into RAW264.7 macrophages. Translocation was detected for both fusions, SseJ-OprF/I-FLAG and SseJ-PcrV-FLAG, by Western blotting using an anti-FLAG antibody (Fig. 1D). Translocation was T3SS2 dependent, since it was not observed in an *ssaV* mutant background (SsaV is an essential component of T3SS2).

### Antibody responses induced by candidate vaccines against P. aeruginosa.

**FIG 2:**
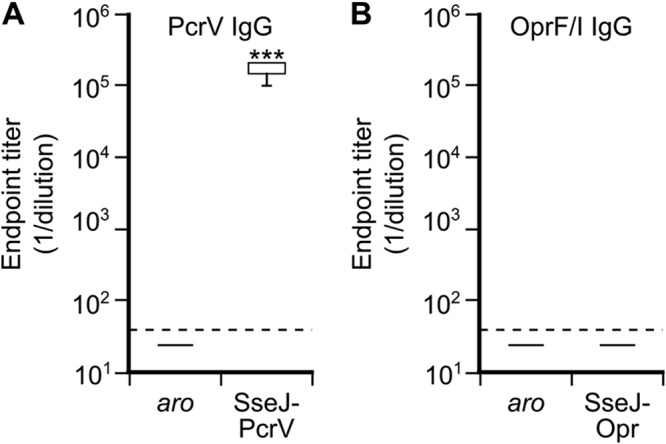
Antibody responses to immunization with *Salmonella* strains expressing *Pseudomonas* antigens. Mice were inoculated intraperitoneally with plasmid-less *S.* Typhimurium *aroA aroB* (*aro*) or with the same *Salmonella* strain carrying pWSK29 derivatives expressing PcrV or Opr fusion proteins as indicated. Serum samples were collected from vaccinated and control mice 21 days after immunization and levels of PcrV (A) or OprF/I (B) specific total IgG were measured by ELISA. Box and whisker plots represent the interquartile ranges and ranges, respectively, and horizontal lines represent median values. ***, *P < *0.001 compared to levels in control mice using the Mann-Whitney U test.

The plasmids generated as described above were introduced into an attenuated strain of *S.* Typhimurium with mutations in *aroA* and *aroB*. Expression of the fusion proteins was detected again in this background, both in LPM (imitating intravacuolar conditions) and during infection of macrophages (Fig. 1E), and C57BL/6 mice were immunized by intraperitoneal injections with the attenuated strains carrying the plasmids. Mice immunized with *Salmonella aroA aroB* without pWSK29 derivatives were used as the controls. Indirect enzyme-linked immunosorbent assays (ELISAs) were performed using sera collected 21 days after immunization. As shown in Fig. 2, immunization with *Salmonella* expressing SseJ-PcrV elicited detectable levels of antigen-specific total IgG in all mice, whereas *Salmonella* expressing SseJ-OprF/I was not able to induce a specific response. As expected, control mice had no detectable antigen-specific IgG.

### Protective responses induced by candidate vaccines.

**FIG 3:**
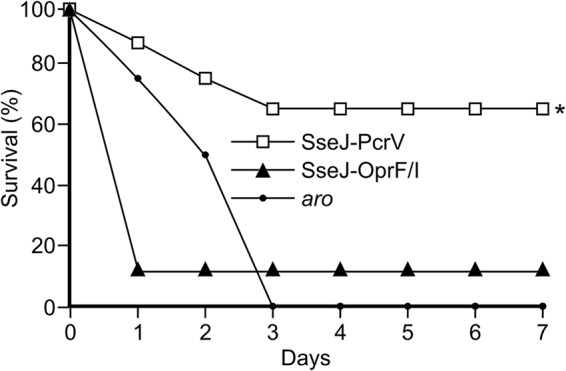
Survival curve of challenged mice. Groups of 8 C57BL/6 mice were intraperitoneally vaccinated with an *aroA aroB* mutant of *S.* Typhimurium (*aro*) or with the same strain carrying the plasmids pIZ2160 or pIZ2267 for expression of the fusion proteins SseJ-OprF/I-FLAG (from the P*sseA* promoter) or SseJ-PcrV-FLAG (from the P*sseA* promoter), respectively. Twenty-one days after the immunization, mice were infected with P. aeruginosa strain PAO1 by intraperitoneal injection, and the survival of mice was monitored for 7 days. Statistical significance was determined by log rank analysis. *, *P < *0.05 compared to levels in control mice inoculated with *Salmonella aroA aroB*.

Vaccine efficacy was tested by infecting immunized and control mice with P. aeruginosa strain PAO1 by intraperitoneal injections, and survival was monitored over 7 days. As shown in Fig. 3, most of the mice vaccinated with *Salmonella* expressing the SseJ-PcrV construct survived the challenge, whereas mice vaccinated with *Salmonella* expressing SseJ-OprF/I or control *Salmonella* were not significantly protected. Statistical analysis of these survival data showed significant protection for the group vaccinated with *Salmonella* expressing SseJ-PcrV, whereas there was no significant difference between the control group and the group vaccinated with *Salmonella* expressing SseJ-OprF/I.

### Effect of vaccination on tissue bacterial loads, postinfection serum cytokine levels, and survival.

**FIG 4:**
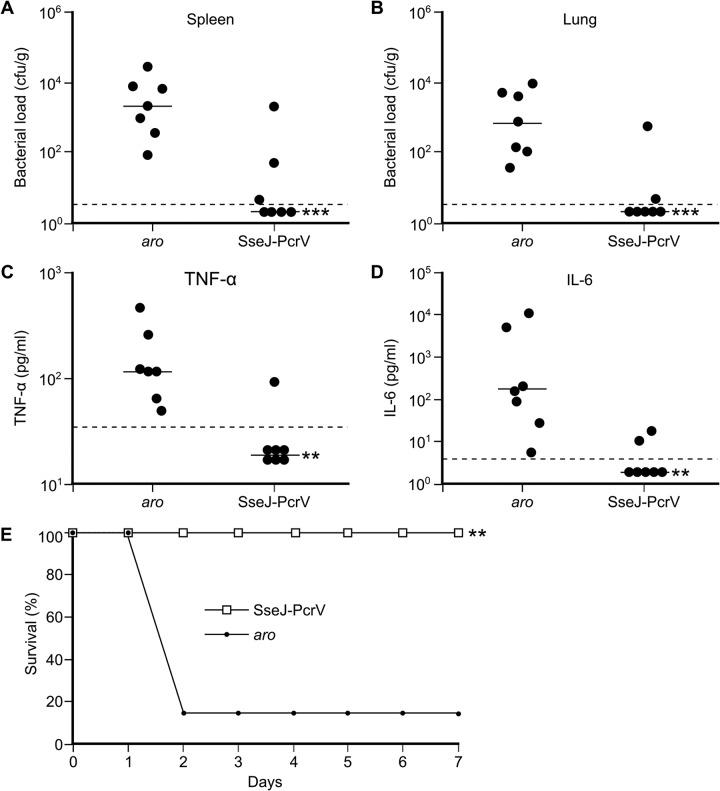
Effect of vaccination on bacterial loads, postinfection proinflammatory cytokine levels, and survival. C57BL/6 mice were intraperitoneally vaccinated with an *aroA aroB* mutant of *S.* Typhimurium (*aro*) or with the same strain carrying the plasmid pIZ2267 for expression of the fusion protein SseJ-PcrV-FLAG from the P*sseA* promoter. Twenty-one days after the immunization, mice were infected with P. aeruginosa strain PAO1 by intraperitoneal injection. Twelve hours postinfection, groups of 7 mice were euthanized to enumerate the spleen (A) and lung (B) bacterial loads and to determine serum levels of TNF-α (C) and IL-6 (D). Data points represent bacterial loads or cytokine levels from individual mice, and horizontal lines represent median values from groups of mice. (E) Survival of other groups of infected mice was monitored for 7 days. **, *P < *0.01; ***, *P < *0.001 compared to levels in control mice inoculated with *Salmonella aroA aroB*. Differences in bacterial loads and cytokine levels were tested using the Mann-Whitney U test, and differences in survival were analyzed by the log rank test.

A new immunization experiment was carried out using the protective vaccine (attenuated *Salmonella* expressing SseJ-PcrV) or attenuated *Salmonella* without a pWSK29 derivative as a control. Immunized and control mice were challenged intraperitoneally with P. aeruginosa PAO1 21 days postimmunization. Twelve hours after infection, spleen and lung bacterial loads were determined (Fig. 4A and B). Vaccination with *Salmonella* expressing the SseJ-PcrV fusion dramatically reduced the number of P. aeruginosa in spleens and lungs. To characterize the effect of immunization on cytokine levels, sera were also collected 12 h after the infection with P. aeruginosa, and the levels of tumor necrosis factor alpha (TNF-α) and interleukin-6 (IL-6) were determined (Fig. 4C and D). Levels of both cytokines were significantly lower in vaccinated mice than in control mice. Finally, the survival of vaccinated mice was monitored for 7 days after challenge with P. aeruginosa (Fig. 4E). All mice vaccinated with *Salmonella* expressing SseJ-PcrV were protected from challenge, whereas 6 of 7 mice inoculated with control *Salmonella* died in less than 48 h.

## DISCUSSION

This study shows that the T3SSs of *Salmonella* can be used as vectors for efficient delivery of P. aeruginosa antigens and elicitation of protective immunity against this opportunistic pathogen. In particular, the expression of a fusion between the *Salmonella* effector SseJ and the *Pseudomonas* antigen PcrV under the control of P*sseA* yielded successful results in the preclinical study shown here. Since P*sseA* responds to intravacuolar signals and SseJ is a specific substrate of the T3SS2, the fusion protein SseJ-PcrV is expected to be expressed by *Salmonella* inside the vacuole of the antigen-presenting cell and translocated from the vacuole into the host cell cytosol. Previous findings suggested that the translocation of fusion proteins results in major histocompatibility complex (MHC) class I- and MHC class II-restricted presentation and that a fusion of the model antigen listeriolysin to SseJ induced T cell responses after vaccination in mice (25). Here, we show that mice immunized with *Salmonella* expressing a fusion of SseJ to PcrV, a relevant P. aeruginosa antigen, produced high levels of specific anti-PcrV IgG and were able to survive an otherwise lethal infection with *Pseudomonas*. Protection was observed in two independent experiments shown in Fig. 3 and Fig. 4E. The differences in the results between these experiments could be due to multiple factors that influence this kind of experiment (differences in the lots of mice that were used, the exact state of the *Salmonella* cultures used for the vaccinations, different *Pseudomonas* cultures used for challenge, reactions of individual mice to intraperitoneal injections, etc.), but importantly, both experiments confirmed that this vaccine conferred statistically significant protection against *Pseudomonas*. The protective effect of this vaccination was confirmed because immunized mice displayed lower bacterial loads in different organs and lower serum levels of proinflammatory cytokines. PcrV was shown previously to induce protective immunity when delivered as a purified recombinant protein antigen, alone or in combination with other antigens (16, 40–43). In a recent report, mice were immunized intraperitoneally with recombinant PcrV formulated with one of three different adjuvants (43). These vaccines were effective in inducing specific immunity against PcrV and in conferring protection against a lethal dose of P. aeruginosa.

Surprisingly, a similar construct with the OprF/I antigen was unable to elicit the production of specific antibodies or to provide protection against *Pseudomonas*. The reasons for the superior immunogenicity of PcrV compared to that of OprF/I in our system are unclear. The recombinant hybrid protein OprF(190-342)/OprI(21-83) was shown to be immunogenic and protective in previous studies (13, 44). Glutathione transferase (GST)-OprF/I elicited specific antibodies against P. aeruginosa and OprF peptides and protected immunodeficient mice against 975-fold the 50% lethal dose of P. aeruginosa, whereas GST alone had no effect (45). A vaccine based on this hybrid protein, IC43, was evaluated in a phase II clinical trial in mechanically ventilated intensive care unit patients, although it did not produce significant differences in infection rates (11).

PcrV is an essential component of the T3SS. This system and its effectors are the major virulence determinants of P. aeruginosa (46). Thus, the T3SS is an appealing target for new therapies (47). PcrV, as part of the translocon, the most accessible part of the T3SS, has been selected for targeting by antibodies developed for clinical use (48). However, few vaccines have been described based on this antigen, presumably because of solubility problems during purification of the protein (41). The methodology employed here circumvents these problems, since the antigen is produced by *Salmonella* and directly translocated through the T3SS2 into the antigen-presenting cells. The use of an *in vivo* inducible promoter, such as P*sseA*, instead of a constitutive promoter is advantageous, because it enhances stable expression and immunogenicity of foreign antigens expressed by *Salmonella* (49). The pWSK29 vector carries the pSC101 replicon that produces 6 to 8 copies per cell (50) and is sufficiently stable for *in vivo* applications (25).

The aim of this study was to characterize the ability of this vaccination approach to produce an antibody response and to protect against infection in a preclinical model. Although this is an important step toward the development of an effective vaccine for the prevention of infections with P. aeruginosa, additional aspects could be optimized in future experiments carried out in this model. (i) Tolerability and toxicity studies would be necessary in order to advance this vaccine candidate. (ii) Other effectors could be tested as carriers for the PcrV antigen, taking into account that the fusion with the highest level of expression *in vitro* is not necessarily the most immunogenic *in vivo* (25). (iii) Since *Salmonella* is also a pathogen, strains used to develop a live vaccine should be attenuated. However, they should also be able to reach, multiply, and persist temporarily in lymphoid organs to stimulate protective immune responses. Thus, a balance between attenuation and immunogenicity is essential for the success of a live vaccine. The *aro* mutants of *S.* Typhimurium used here are auxotrophic for aromatic amino acids and several essential vitamins. These mutants are safe and immunogenic in mouse models (51), where this serovar can reach systemic organs, and have previously been used as carriers of heterologous antigens (52). Since *S.* Typhimurium causes only self-limited intestinal disease in immunocompetent humans and does not reach systemic sites in this host, the human-restricted S. enterica serovar Typhi is also being tested as a live vector for humans (53). A double *aro* mutant of *S.* Typhi is highly immunogenic, but additional attenuation could be desirable in humans; therefore, other means of enhancing vaccine efficacy and safety have been proposed (54). In particular, the safety of live attenuated vaccines needs to be determined in immunocompromised individuals, since they represent a relevant target for vaccination against *Pseudomonas*. In fact, an *aro* mutant may be virulent in immunodeficient mice (55), although moderate immunosuppression, causing increased susceptibility to wild-type *Salmonella,* does not affect resistance to an *aroA Salmonella* vaccine strain (56). Other attenuating mutations or combinations of mutations could be used to increase safety (55). (iv) Another aspect to be considered for future development of this vaccine is the route of immunization. The intraperitoneal injection is employed in many preclinical studies but is not typically used in the clinical setting as a route of immunization. However, mice can also be inoculated with *Salmonella* via the intravenous, subcutaneous, oral, intranasal, and rectal routes (57, 58), and some of these protocols should be tested in animal models before progressing into the clinical stages. It is also worth noting that the cell-mediated immune response was not evaluated in this study, an aspect that could be further characterized in future studies.

In conclusion, this study shows that the SPI2-related T3SS of *Salmonella* is an appropriate vehicle to deliver the *Pseudomonas* antigen PcrV and to generate a protective live vaccine against this opportunistic pathogen.

## MATERIALS AND METHODS

### Bacterial strains, bacteriophages, and strain construction.

Bacterial strains used in this study are described in Table 1. *S.* Typhimurium strains were derived from the wild-type strain ATCC 14028. Transductional crosses using phage P22 HT 105/1 *int201* (59) were used for strain construction (60).

**TABLE 1:** Bacterial strains and plasmids used in this study

Strain or plasmid	Relevant characteristics	Source or reference
Strains		
Escherichia coli		
DH5α	*supE44* Δ*lacU169* (φ80 *lacZ*ΔM15) *hsdR17 recA1 endA1 gyrA96 thi-1 relA1*	67
BL21(DE3)	F^−-^ *ompT gal dcm lon hsdS*_B_(r^−^ m^−^; E. coli B strain), with DE3, a λ prophage carrying the T7 RNA *pol* gene	Stratagene
Pseudomonas aeruginosa		
PAO1	Reference strain	68
Salmonella enterica serovar Typhimurium^*a*^		
14028	Wild type	ATCC
SV4338	*aroA551*::Tn*10*	Laboratory stock
SV5136	*ssaV*::Cm	Laboratory stock
SV8462	Δ*aroB*::Km	Laboratory stock
SV9699	*aroA551*::Tn*10* Δ*aroB*::Km	This work
Plasmids		
pET15b	6×His fusion vector, Ap^r^	Novagen
pET15b-OprF/I	OprF/I cloned with NdeI and BamHI	This work
pGEX-4T-2	GST fusion vector, Ap^r^	GE Healthcare
pIZ2160	pWSK29-PsseA-SseJ-OprF/I-FLAG	This work
pIZ2267	pWSK29-PsseA-SseJ-PcrV-FLAG	This work
pIZ2338	pGEX-4T-2-PcrV	This work
pWSK29	Low-copy-number vector, Ap^r^	50

a Derivatives of these strains were used as indicated in the text.

### Bacterial culture.

The standard culture medium for all bacteria was Luria-Bertani (LB) broth. For SPI2-inducing conditions, bacteria were inoculated in LPM at pH 5.8 (61) and incubated overnight at 37°C with shaking. The following supplements were added to LPM to allow growth of *aro* strains: Phe, 40 μg/ml; Trp, 40 μg/ml; Tyr, 40 μg/ml; *p*-aminobenzoate, 10 μg/ml; 2,3-dihydroxybenzoate, 10 μg/ml. Solid medium contained 1.5% agar. Antibiotics were used at previously described concentrations (62).

### DNA amplification by PCR and sequencing.

Amplification reactions were carried out in a T100 thermal cycler (Bio-Rad) using KAPA HiFi DNA polymerase (Kapa Biosystems) or MyTaq Red DNA polymerase (Bioline) according to the instructions of the suppliers. The oligonucleotides are described in Table 2. Constructs were sequenced with an automated DNA sequencer (Stab Vida, Oeiras, Portugal).

**TABLE 2:** Oligonucleotides used in this study

Oligonucleotide	Sequence 5′→3′
Amplification of *pcrV* with FLAG tag for cloning in pWSK29	
pcrVfwBam	ATCGGGATCCGAAGTCAGAAACCTTAATGC
pcrVFLAGrevNot	GCATGCGGCCGCCTATTTATCGTCGTCATCTTTGTAGTCGATCGCGCTGAGAATGTCGC
Fusion of *oprF* and *oprI* with FLAG tag	
oprFfwBam	ATCGGGATCCGCTCCGGCTCCGGAACCGGTTGCCGAC
oprFrev	TTCAACGCGACGGTTGATAGCGCG
oprF/oprIfw	GAAGGCCGCGCTATCAACCGTCGCGTTGAAAGCAGCCACTCCAAAGAAAC CGAAGCT
oprIFLAGrevNot	GCATGCGGCCGCCTATTTATCGTCGTCATCTTTGTAGTCCTTGCGGCTGGCTTTTTCCAG
Amplification of *sseJ*	
sseJfwEco	CCTAGAATTCGTAAGGAGGACACTATGCC
sseJrevBam	ACGTGGATCCTTCAGTGGAATAATGATGAG
Amplification of *sseA* promoter	
PsseAfwKpn	GCTAGGTACCAGAAGAGAACAACGGCAAG
PsseArevEco	CACTGAATTCACGATAGATAATTAACGTGC
Amplification of *pcrV* for cloning in pGEX-4T-2	
pcrVfwBam	ATCGGGATCCGAAGTCAGAAACCTTAATGC
pcrVrevEco	ATCGGAATTCCTAGATCGCGCTGAGAATGT

### Plasmids.

The plasmids used in this study are listed in Table 1. The construction of derivatives of pWSK29 for vaccine assays was carried out in three steps (Fig. 1): (i) cloning of P. aeruginosa DNA fragments encoding antigens PcrV and OprF(190-342)/OprI(21-83) in fusion with FLAG using BamHI and NotI restriction sites; (ii) addition of *S.* Typhimurium DNA encoding effector SseJ using EcoRI and BamHI restriction sites; (iii) addition of P*sseA* using KpnI and EcoRI. The hybrid gene *oprF-oprI* was generated using the gene splicing by overlap extension approach (63) as previously described (45) using oligonucleotides shown in Table 2. To generate plasmid pIZ2338, the coding region of *pcrV* was amplified by colony PCR using lysed P. aeruginosa PAO1 as the template and cloned into pGEX-4T-2 using BamHI and EcoRI restriction sites. To generate plasmid pET15b-OprF/I, *oprF-oprI* was first synthetized *in vitro* (GenScript) and then transferred from the original vector (pUC57) into pET15b using NdeI and BamHI restriction enzymes.

### Expression and purification of recombinant proteins in E. coli.

Plasmids pET15b-OprF/I, expressing 6×His-OprF/I, or pIZ2338, expressing GST-PcrV, were transformed into E. coli BL21(DE3). Bacteria were grown in LB with ampicillin at 37°C. At mid-exponential-growth phase, 1 mM IPTG (isopropyl-β-d-thiogalactopyranoside) was added to induce expression, and incubation was pursued for 4 h. The cells were harvested by centrifugation, and the cell pellet was resuspended in NP-40 lysis buffer (64). A commercial mixture of protease inhibitors (Sigma-Aldrich) was added to the suspension, and lysis was performed by sonication. The cell lysate was centrifuged at 15,000 × *g* for 30 min at 4°C, and the fusion proteins were isolated from the supernatants by affinity chromatography with Ni-nitrilotriacetic acid (NTA) agarose beads (Qiagen) or glutathione agarose beads (Sigma-Aldrich) according to the manufacturers’ protocols.

### Mammalian cell culture.

RAW264.7 cells (murine macrophages, ECACC number 91062702) were cultured in Dulbecco’s modified Eagle’s medium (DMEM) supplemented with 10% fetal calf serum and 2 mM l-glutamine. Penicillin (60 μg/ml) and streptomycin (100 μg/ml) were included in the culture medium (except for bacterial infection experiments). Cells were maintained in a 5% CO_2_ humidified atmosphere at 37°C.

### Bacterial infection of cultured cells and protein translocation assays.

RAW264.7 cells were plated 24 h before infection in 6-well plates at 8 × 10^5^ cells per well and incubated 24 h at 37°C with 5% CO_2_ in medium without antibiotics. Bacteria carrying plasmids expressing FLAG fusions were grown in LB medium with ampicillin for 24 h at 37°C with shaking, were added to the cell monolayers at a multiplicity of infection of 250 bacteria/cell, and then were incubated at 37°C with 5% CO_2_. The cell culture was washed twice with phosphate-buffered saline (PBS) 1 h postinfection, overlaid with DMEM containing 100 μg/ml gentamicin and ampicillin, and incubated for 1 h. The culture was then washed twice with PBS, covered with DMEM with gentamicin (16 μg/ml) and ampicillin (100 μg/ml), and incubated for 6 h. To study the translocation of FLAG fusions, infected mammalian cells were lysed with 1% Triton X-100. Cell lysates were processed for electrophoresis and Western blotting as previously described (65). Anti-DnaK (1:5,000; Assay Designs) and anti-GroEL (1:20,000; Sigma-Aldrich) antibodies were used as loading or contamination controls.

### Analysis of expression of fusion proteins.

*Salmonella* strains carrying plasmids expressing FLAG fusions were grown overnight in LB medium at 37°C with shaking. Bacteria were then washed, diluted 1:50 in LPM, and incubated at 37°C with shaking for 8 h. The bacteria were then pelleted by centrifugation and lysed by boiling in Laemmli sample buffer. Proteins were separated by SDS-PAGE and analyzed by immunoblotting with a monoclonal antibody directed to the FLAG epitope (1:10,000; Sigma-Aldrich). Goat anti-mouse horseradish peroxidase (HRP)-conjugated antibody (1:5,000; Bio-Rad) was used as the secondary antibody.

### Mouse immunization and infection with P. aeruginosa.

Mice were maintained in the IBiS facility, and their care was in accordance with institutional guidelines. Attenuated *S.* Typhimurium strain SV9699 (*aroA551*::Tn*10* Δ*aroB*::Km) (Table 1) with appropriate plasmids was grown overnight at 37°C with shaking in LB with ampicillin, diluted in fresh medium (1:100), and grown to an optical density at 600 nm (OD_600_) of 0.3 to 0.6. Vaccination was carried out in 6- to 8-week-old female C57BL/6 mice (Charles River Laboratories) by a single intraperitoneal injection with 0.2 ml of PBS containing 2 × 10^5^ CFU of *Salmonella*. Mice were infected with P. aeruginosa strain PAO1 on day 21 postimmunization by intraperitoneal injection with 9 × 10^6^ bacteria (3.6× the 50% lethal dose [LD_50_]) in 0.2 ml of PBS, and survival was monitored for 7 days. Mice that received the same infection were housed together with up to 5 mice per cage. Mice were monitored twice daily and culled using thiopental at the end of the experiments. The procedures (intraperitoneal injections) made the use of analgesics unnecessary. To minimize animal suffering, euthanasia using thiopental was carried out immediately when detecting severe clinical signs: hunching, labored breathing, severe weight loss, inactivity, or lethargy. Experiments involving the use of animals were approved by the Committee on Ethics and Experimentation of the Consejería de Agricultura, Pesca y Desarrollo Rural (Junta de Andalucía, Spain) (permit number 18-01-16-005) and were in accordance with the EU Directive 2010/63/EU for animal experiments.

### Spleen and lung bacterial loads and serum cytokine levels.

Postinfection bacterial loads were determined in vaccinated and control mice 12 h after infection. Mice were euthanized with thiopental, and after the collection of blood samples from the retro-orbital sinus, spleens and lungs were aseptically removed, weighed, and homogenized in 2 ml of physiological saline. Serial log dilutions were plated on agar plates for bacterial quantification. Serum levels of tumor necrosis factor alpha (TNF-α) and interleukin-6 (IL-6) were determined in mice at 12 h postinfection using DuoSet ELISA kits (R&D Systems).

### Enzyme-linked immunosorbent assays.

For indirect ELISAs, 96-well plates were coated with 100 μl per well of 1-μg/ml solutions of purified 6×His-OprF/I or GST-PcrV by incubating at 4°C overnight. ELISAs were performed using sera collected on day 21 as described previously (66). Antibody titers were measured against the OprF/I or PcrV antigens and were defined as the dilution in which spectrophotometric readings were at least 0.1 units greater than that in the background wells (wells containing no serum).

### Statistical analysis.

Antibody titers, bacterial loads, and cytokine levels were compared using the Mann-Whitney U test. Survival data were compared using the log rank test. Statistics were performed using SPSS version 24.0 software (SPSS Inc.). *P* values of 0.05 or less were considered significant.
